# Causal Effect and Personalization of Intraoperative Hypotension Burden on Postoperative Acute Kidney Injury: A Doubly Robust Analysis of the VitalDB Cohort

**DOI:** 10.3390/jpm16070371

**Published:** 2026-07-10

**Authors:** Seung-Bo Lee

**Affiliations:** Department of Medical Informatics, Keimyung University School of Medicine, Daegu 42601, Republic of Korea; koreateam23@gmail.com

**Keywords:** intraoperative hypotension, acute kidney injury, causal inference, doubly robust estimation, treatment effect heterogeneity, causal forest, personalized medicine, risk prediction, VitalDB

## Abstract

**Background**: Intraoperative hypotension (IOH) is the leading modifiable contributor to postoperative acute kidney injury (AKI), yet most evidence is associational and the heterogeneity of its effect is unknown. We estimated the causal effect of IOH burden on AKI and tested whether the most susceptible patients can be identified preoperatively. **Methods**: In a retrospective cohort of 2726 general-anesthesia cases from VitalDB, the exposure was the time-integrated mean arterial pressure (MAP) <65 mmHg burden (≥30, ≥60 and ≥120 mmHg·min) and the outcome was KDIGO-defined AKI within 7 days. The primary estimator was pre-treatment-adjusted augmented inverse-probability weighting (AIPW; doubly robust) with bootstrap 95% confidence intervals (CIs). Sensitivity analyses comprised a controlled-direct-effect model, negative control outcomes, E-values and vasopressor-stratified estimates. Effect heterogeneity was estimated with a causal forest; preoperative gradient-boosted models and decision-curve analysis assessed personalization and clinical utility. **Results**: AKI occurred in 205 (7.52%) cases. At 60 mmHg·min the AIPW risk difference was +3.00 percentage points (pp; 95% CI +0.84 to +5.26), with a monotonic dose–response (+2.78 to +7.62 pp across thresholds) and E-values rising from 2.08 to 3.44. The effect was concentrated in patients with elevated preoperative creatinine (conditional effect +9.86 pp, more than twice the cohort average). This susceptibility was recoverable from routine preoperative variables alone, with intraoperative waveform features conferring no measurable improvement (ΔAUROC −0.001). For predicting AKI itself, a parsimonious 4-feature preoperative score matched a 27-feature model (AUROC 0.775 vs. 0.768) and provided positive net benefit. **Conclusions**: Intraoperative hypotension burden shows a dose-dependent association with postoperative AKI that is consistent with a causal effect, concentrated in patients with reduced baseline renal reserve who are identifiable from routine preoperative data without intraoperative waveform infrastructure.

## 1. Introduction

Postoperative acute kidney injury (AKI) is among the most common complications of major non-cardiac surgery and is strongly associated with chronic kidney disease, cardiovascular events, prolonged hospitalization and death [1,2]. In a contemporary cohort of 13,790 elective major non-cardiac operations, AKI occurred in 9.7% of patients and carried an approximately eight-fold increase in the odds of in-hospital death, with low preoperative albumin, hemoglobin and glomerular filtration rate among the strongest baseline correlates [3]. Intraoperative hypotension (IOH) is the most prominent potentially modifiable contributor to this risk [4,5]. Yet, susceptibility to hypotensive renal injury is unlikely to be uniform: patients differ in baseline renal reserve, and the clinically decisive question is therefore not only whether IOH causes AKI on average, but for whom intraoperative blood-pressure protection would matter most.

Two gaps limit the existing evidence. First, although IOH is among the most studied perioperative exposures, it lacks a standard definition: one systematic review identified 140 definitions across 130 studies, with reported incidences ranging from 5% to 99% [6], and consensus statements now favor mean arterial pressure (MAP) thresholds of 60–70 mmHg, with even brief exposures associated with organ injury [7]. More fundamentally, most reports rest on multivariable-adjusted associations rather than explicit causal estimands, and the role of intraoperative vasopressors—which are both a response to hypotension and a possible independent nephrotoxic exposure—remains entangled as a simultaneous confounder and mediator [8]. Second, prior work characterizes average effects and average risk: established preoperative indices predict the occurrence of AKI [9] but not the heterogeneity of the treatment effect itself, leaving unanswered which patients would derive the greatest benefit from tighter intraoperative MAP control.

We addressed both gaps in a high-resolution, publicly reproducible cohort. Using the VitalDB biosignal database, which provides beat-to-beat invasive arterial waveforms alongside routine perioperative and laboratory data, we (i) estimated the causal effect of intraoperative hypotension burden on 7-day postoperative AKI using a doubly robust augmented inverse-probability-weighting framework with pre-specified sensitivity analyses (E-value, negative control outcomes, and mediator-adjusted and vasopressor-stratified models), and (ii) characterized effect heterogeneity with a causal forest and tested whether the patients most susceptible to hypotensive renal injury could be identified from routine preoperative variables alone. We hypothesized that IOH burden raises AKI risk in a dose-dependent manner, that this effect is concentrated in patients with reduced baseline renal reserve, and that such high-susceptibility patients are identifiable before incision without intraoperative waveform infrastructure.

## 2. Materials and Methods

### 2.1. Study Design and Data Source

We conducted a retrospective cohort study using the VitalDB biosignal database (release v1.0.0), a publicly accessible high-resolution intraoperative dataset from Seoul National University Hospital comprising 6388 surgical cases [10]. The database provides time-synchronized vital sign waveforms (including invasive arterial blood pressure), 73 perioperative clinical parameters and 34 time-series laboratory results, each indexed in seconds relative to anesthesia start. Reporting follows the STROBE statement for observational studies [11] and the TRIPOD+AI extension for the prediction-model component [12]. As VitalDB is fully de-identified and publicly distributed under the PhysioNet Credentialed Health Data License, no separate ethics approval was sought.

### 2.2. Eligibility and Cohort Construction

**Figure 1:**
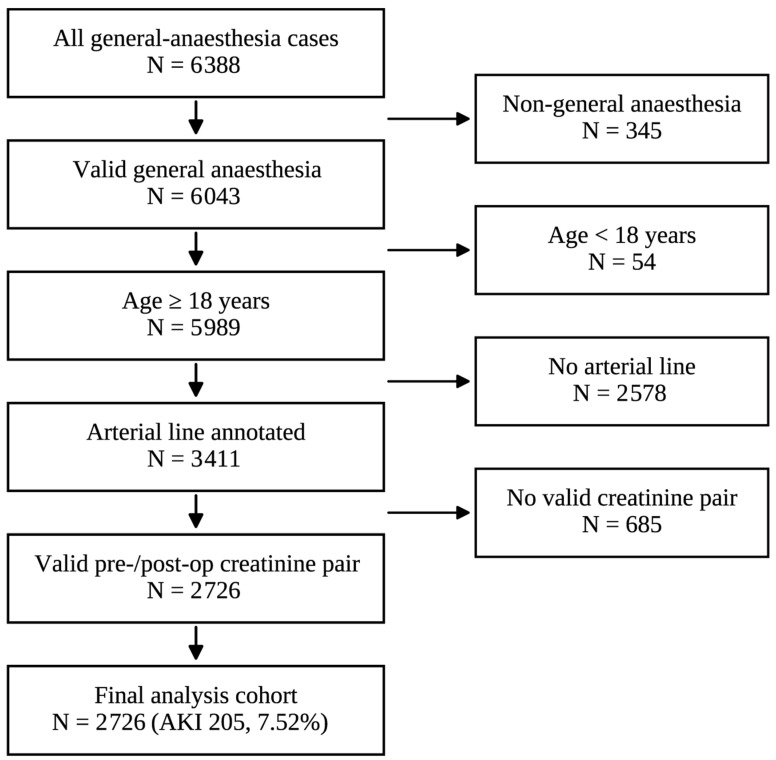
Cohort selection flow diagram. Sequential exclusion of cases from VitalDB v1.0.0 (*n* = 6388) yielded the final analysis cohort of 2726 cases with valid pre- and postoperative creatinine pairs. Postoperative AKI occurred in 205 cases (7.52%).

From 6388 cases in VitalDB v1.0.0, we sequentially excluded 345 non-general-anesthesia cases, 54 cases in patients aged < 18 yr, 2578 cases lacking annotated invasive arterial line monitoring (required for MAP integration), and 685 cases without a valid preoperative serum creatinine paired with at least one postoperative creatinine measurement within 7 days. The final analysis cohort comprised 2726 cases (Figure 1).

### 2.3. Exposure

The exposure was the intraoperative hypotension burden, operationalized as the time-integrated area below a MAP threshold of 65 mmHg (mmHg·min), computed from the invasive arterial waveform sampled at 100 Hz. Three pre-specified burden thresholds were analyzed in parallel—≥30, ≥60 and ≥120 mmHg·min—representing increasing dose levels. We anchored the exposure at MAP = 65 mmHg because it is the most extensively validated and guideline-endorsed harm threshold for perioperative hypotension: large cohorts report a dose-dependent rise in acute kidney and myocardial injury below this level [4,5], the incidence and apparent effect of intraoperative hypotension are strongly dependent on the chosen definition [6], and current consensus guidance recommends maintaining MAP ≥ 65 mmHg in general adult patients [7]. Concretely, for each case the burden B (in mmHg·min) was the time integral of the mean arterial pressure deficit below 65 mmHg over the operative interval [0, T], computed on the arterial waveform after excluding artefactual values outside 20–250 mmHg:

*B* = (1/60) ∫_0_^T^ max(0, 65 − MAP(*t*)) d*t*

A case was classified as exposed when B reached the pre-specified threshold (30, 60, or 120 mmHg·min).

### 2.4. Outcome

The primary outcome was postoperative AKI within 7 days, defined per the KDIGO Clinical Practice Guideline [13] as either an absolute increase in serum creatinine of ≥0.3 mg/dL within any 48 h window or a ≥1.5-fold relative increase from the most recent preoperative creatinine. Owing to the absence of reliable hourly urine-output records in VitalDB, AKI was defined on serum creatinine criteria only, corresponding to KDIGO Stage 1 or higher.

### 2.5. Covariates

Pre-treatment covariates included age, sex, height, weight, ASA physical status, emergency-operation status, surgical department and preoperative laboratory values (hemoglobin, platelet count, sodium, potassium, blood urea nitrogen, creatinine, albumin, PT, aPTT), with comorbidity flags for hypertension and diabetes mellitus. The primary analysis adjusted for these pre-treatment covariates only. Perioperative covariates (intraoperative fluid volume, vasopressor administration, anesthesia duration) lie downstream of the exposure and were used only in a controlled-direct-effect sensitivity analysis.

### 2.6. Causal Estimands and Estimators

We targeted the average treatment effect, expressed as the counterfactual risk difference in AKI between scenarios in which all patients did versus did not exceed the IOH burden threshold. At each threshold we computed four estimators: the unadjusted crude risk difference; parametric G-computation; inverse probability of treatment weighting (IPTW) with stabilized weights; and augmented inverse-probability weighting (AIPW), combining outcome and propensity models in a doubly robust manner [14,15]. The primary AIPW analysis adjusted for pre-treatment covariates only, estimating the total causal effect; primary 95% CIs were obtained from a non-parametric bootstrap (1000 resamples).

### 2.7. Sensitivity Analyses

Four pre-specified sensitivity analyses addressed distinct threats to validity: a perioperative-covariate-augmented AIPW estimating the controlled direct effect; negative control outcome (NCO) analyses using hyponatremia and hyperglycemia [16]; E-value computation quantifying the minimum unmeasured-confounder strength required to nullify the effect [17]; and vasopressor-stratified AIPW estimated separately in the no-vasopressor and vasopressor strata.

### 2.8. Heterogeneity and Internal Validation

Conditional average treatment effects (CATEs) at 60 mmHg·min were estimated with a causal forest (generalized random forest) [18,19], with subgroup CATEs for pre-specified strata. Three CATE prediction models were trained on a 70% split: a gradient-boosted machine on 27 preoperative tabular features (M1); the same extended with 20 intraoperative waveform features (M2); and a multi-layer perceptron on the combined set (M3). Beyond random splitting, temporal and leave-one-department-out validation was applied, and decision-curve analysis (DCA) compared net benefit across decision thresholds of 1–40% [20]. Analyses used Python 3.11 (scikit-learn, econml, matplotlib); the pipeline is reproducible from the public VitalDB dataset.

## 3. Results

### 3.1. Cohort Characteristics

Of 6388 cases, 2726 met all eligibility criteria (Figure 1). Postoperative AKI occurred in 205 cases (7.52%). Patients meeting the 60 mmHg·min threshold (*n* = 406, 14.9%) were modestly older (median 63 [IQR 53–72] vs. 61 [52–69] yr) and more frequently male (67.7% vs. 57.9%), with lower preoperative hemoglobin (12.1 vs. 13.0 g/dL) and albumin (3.90 vs. 4.20 g/dL). Crude AKI incidence was 20.0% in the exposed versus 5.3% in the unexposed group (Table 1).

### 3.2. Causal Effect of IOH Burden on AKI

Risk-difference estimates differed markedly across estimators (Table 2). At 60 mmHg·min the crude risk difference (+14.61 pp) substantially overstated the effect once confounding was addressed. The primary pre-treatment-only AIPW yielded an AKI risk increase of +3.00 pp (bootstrap 95% CI +0.84 to +5.26). The controlled-direct-effect model gave +2.24 pp, consistent with partial mediation through intraoperative fluid and vasopressor management. IPTW gave intermediate values (+7.68 pp) and G-computation the most conservative (+0.72 pp).

**Figure 2:**
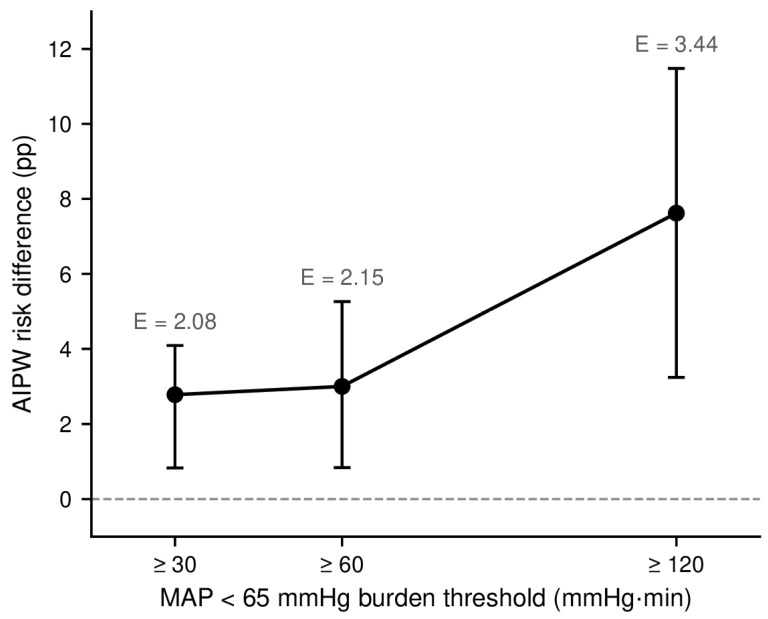
Dose–response relationship between intraoperative hypotension burden and postoperative AKI. AIPW risk-difference estimates (shown as black filled circles) with 95% CI are plotted against MAP < 65 burden thresholds; E-values are annotated at each point.

A clear dose–response relationship was observed (Figure 2): primary AIPW risk differences increased monotonically from +2.78 pp at ≥30 mmHg·min to +3.00 pp at ≥60 and +7.62 pp at ≥120 mmHg·min, with corresponding E-values rising from 2.08 to 2.15 to 3.44.

### 3.3. Sensitivity Analyses

The controlled-direct-effect model gave smaller risk differences than the primary total effect across all thresholds, as expected when adjusting for mediators (Supplementary Table S1). Among negative control outcomes (Supplementary Table S2), hyponatremia yielded a null association (RD +1.75 pp, 95% CI −1.02 to +4.51 at 60 mmHg·min), whereas hyperglycemia yielded a positive association (+5.16 pp, +2.17 to +8.15); the latter indicates that some residual surgical-severity confounding cannot be excluded, bounded by the E-value (Supplementary Table S3). The positive association held in both the no-vasopressor (+5.02 pp) and vasopressor (+3.54 pp) strata (Supplementary Table S4). All sensitivity estimates are summarized in Supplementary Figure S1.

### 3.4. Heterogeneity of Treatment Effect

**Figure 3:**
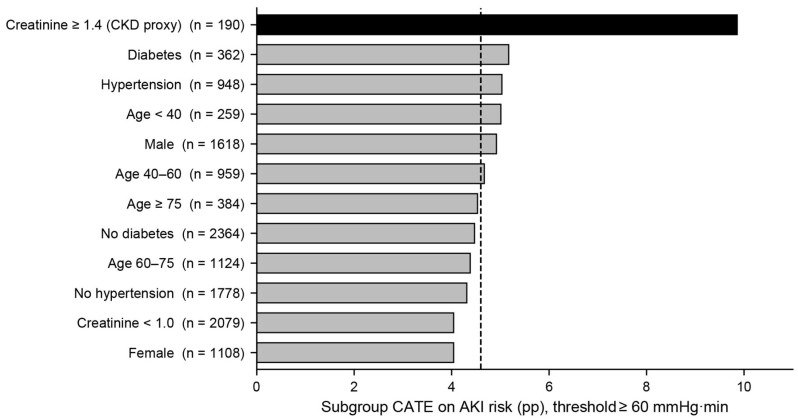
Heterogeneous treatment effects across pre-specified subgroups (causal forest, threshold 60 mmHg·min). The dashed line marks the overall CATE (+0.046); patients with elevated preoperative creatinine show the largest effect (+9.86 pp).

Causal forest subgroup estimates revealed substantial heterogeneity (Figure 3). The strongest signal was in patients with elevated preoperative creatinine (≥1.4 mg/dL, a CKD proxy), whose conditional effect was +9.86 pp—more than twice the cohort-average causal-forest CATE of +4.6 pp (and roughly three-fold the primary AIPW total effect of +3.00 pp). Smaller increments were seen in diabetes (+5.17 pp) and hypertension (+5.03 pp); age- and sex-stratified effects varied within +4.0 to +5.0 pp, indicating that preoperative renal function is the dominant modifier.

### 3.5. Prediction Performance and Internal Validation

The 27-feature preoperative GBM (M1) reproduced the causal-forest-derived CATE with RMSE 0.0067, Spearman ρ 0.860 and R^2^ 0.794, and discriminated the top-decile high-CATE subset with an AUROC of 0.977 (Supplementary Table S5; predicted versus true agreement in Supplementary Figure S2). This quantifies internal recovery of the model-defined CATE target rather than prediction of AKI occurrence, which is reported separately below. Adding intraoperative waveform features (M2) produced no measurable improvement (ΔAUROC −0.001, ΔR^2^ −0.005; Supplementary Figure S3), and the MLP variant performed slightly worse. Feature importance was dominated by preoperative albumin (0.253), creatinine (0.173) and hemoglobin (0.080) (Supplementary Table S7). Temporal hold-out (top-10% AUROC 0.987) and leave-one-department-out validation (AUROC ≥ 0.93 across all four departments) confirmed stability (Supplementary Table S6). Together with the null contribution of waveform features, this indicates that personalized susceptibility is a preoperatively fixed trait reflecting baseline renal reserve. Accordingly, the AUROC of 0.977 should not be read as evidence of clinical readiness; discrimination for the observed AKI outcome—the clinically relevant benchmark—is reported in Section 3.6 (Table 3, AUROC 0.768).

### 3.6. Comparison with Existing Risk Scores and Decision-Curve Analysis

In the held-out test set, AUROC for predicting actual postoperative AKI—as distinct from the model-defined CATE target above—was 0.580 for preoperative creatinine alone, 0.741 for ASA-PS alone, 0.775 for a four-feature logistic model and 0.768 for the 27-feature CATE GBM (Table 3); the four-feature model was statistically indistinguishable from the full model, indicating a performance plateau.

**Figure 4:**
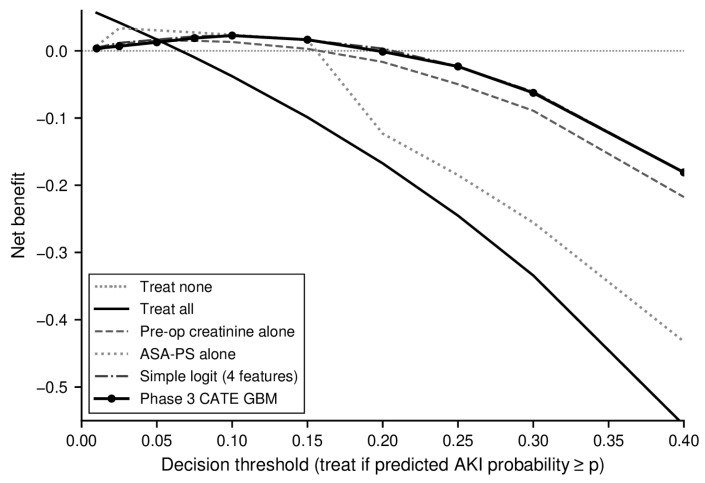
Decision-curve analysis comparing net clinical benefit of four preoperative AKI risk-stratification strategies across decision thresholds.

Decision-curve analysis (Figure 4) showed that both the four-feature model and the CATE GBM provided positive net benefit over a treat-none strategy across the 2.5–15% decision-threshold range, exceeding treat-all from approximately 7.5% upward, with the two model curves nearly indistinguishable.

## 4. Discussion

In this doubly robust causal analysis of 2726 general-anesthesia cases from VitalDB, surpassing an intraoperative MAP < 65 mmHg burden of 60 mmHg·min was associated with a pre-treatment-adjusted increase in 7-day AKI risk of 3.00 pp (bootstrap 95% CI +0.84 to +5.26), following a monotonic dose–response (+2.78 to +7.62 pp) with E-values rising from 2.08 to 3.44. The effect was strongly heterogeneous: patients with elevated preoperative creatinine had a conditional effect of +9.86 pp—more than twice the cohort average—whereas age- and sex-defined strata varied little. A 27-feature preoperative model recovered these high-susceptibility patients with an AUROC of 0.977, and adding intraoperative waveform features yielded no improvement (ΔAUROC −0.001), indicating a preoperatively fixed trait dominated by baseline renal reserve.

Our findings both corroborate and extend the existing literature. Sun and colleagues reported that sustained MAP < 55 mmHg conferred an adjusted AKI odds ratio of 2.34 for 11–20 min and 3.53 beyond 20 min [4], and a recent systematic review confirmed dose-dependence in non-cardiac surgery [21]. The most directly comparable work, a 38,338-patient cohort, found that both the area under a MAP of 65 mmHg and the cumulative intraoperative norepinephrine dose were independently associated with AKI [8]. We addressed this directly: the positive association persisted in both the no-vasopressor (+5.02 pp) and vasopressor (+3.54 pp) strata, and the controlled-direct-effect model attenuated but did not abolish the effect (+2.24 pp), quantifying the mediated fraction. The fact that the effect was at least as large in the no-vasopressor stratum indicates that the association is not driven solely by vasopressor exposure; it does not imply a protective vasopressor effect, which this observational design cannot establish. More generally, because vasopressors act simultaneously as a response to hypotension and as a potential independent contributor to renal outcomes—treatment-confounder feedback that no observational analysis can fully resolve—our controlled-direct-effect and vasopressor-stratified estimates should be read as bounding this influence rather than isolating it.

Methodologically, whereas most prior IOH–AKI evidence rests on multivariable-adjusted associations, we triangulated four estimators under a potential-outcomes framework. The crude risk difference (+14.61 pp) collapsed to +3.00 pp once confounding was addressed by doubly robust AIPW [14,15]. Robustness to unmeasured confounding was formalized with E-values [17], which exceeded the conventional threshold of 2 at the 60 and 120 mmHg·min levels. Negative control outcomes [16] provided a candid internal check: a null association with hyponatremia argues against generalized residual confounding, whereas a positive association with hyperglycemia signals shared upstream surgical-severity pathways—reported transparently rather than as confirmation. Heterogeneity was estimated with a causal forest [18,19] and clinical utility with decision-curve analysis [20].

Clinically, these results refine where intraoperative blood-pressure protection is likely to matter most. The INPRESS trial demonstrated that individualized blood-pressure management reduced postoperative organ dysfunction, including renal injury, in high-risk patients [22], and international consensus now supports structured perioperative arterial-pressure management [23]. Our heterogeneity analysis suggests that patients with reduced baseline renal reserve—identifiable before incision—stand to gain disproportionately. Because the personalization signal was carried almost entirely by routine preoperative laboratory values and required no intraoperative waveform infrastructure, it could be implemented as a single electronic-health-record function. A parsimonious 4-feature score performed indistinguishably from the 27-feature model (AUROC 0.775 vs. 0.768), supporting the simpler instrument.

Several limitations temper these conclusions. The cohort derives from a single tertiary center, with general surgery comprising the majority of cases, so external validity is unproven. AKI was ascertained from serum creatinine alone, as reliable hourly urine output is unavailable in VitalDB; this may miss purely oliguric or early transient AKI, and, because creatinine rises only after injury has occurred, the outcome reflects the occurrence rather than the precise timing of AKI. Such outcome misclassification is expected to be non-differential with respect to hypotension exposure and would bias the causal estimate toward the null, so the reported effect is more likely conservative than inflated. Exposure was defined by a fixed, population-level absolute threshold (MAP < 65 mmHg); because individual patients—for example those with long-standing hypertension—may require a higher perfusion pressure, this does not capture inter-individual differences in hypotension tolerance. Our heterogeneity analysis partly mitigates this by identifying subgroups (elevated preoperative creatinine, chronic hypertension) with a larger causal effect, but an individualized or baseline-relative exposure definition and prospective evaluation of personalized dynamic MAP targets such as those tested in INPRESS remain important extensions. Residual confounding cannot be excluded, and the positive hyperglycemia negative control signal indicates some surgical-severity confounding may persist despite doubly robust adjustment; accordingly, the evidence against unmeasured confounding is strongest at the ≥120 mmHg·min threshold, where the baseline-adjusted E-value and its lower confidence bound both exceed 2, and is weaker at the lower thresholds. Finally, the conditional effects used to train the prediction models were derived from a causal forest rather than an external ground truth, so the reported discrimination reflects internal recovery of model-defined targets and should be interpreted as a feasibility signal pending prospective validation.

## 5. Conclusions

Within these constraints, our analysis provides doubly robust, dose-dependent and sensitivity-tested evidence that intraoperative hypotension burden is associated with postoperative AKI in a manner consistent with a causal effect, and that the patients who would benefit most from its prevention can be identified from routine preoperative data alone. Future work should pursue multicenter external validation and a prospective trial testing whether CATE-guided, renal-reserve-targeted intraoperative blood-pressure management reduces AKI.

## Figures and Tables

**Table 1:** Cohort characteristics stratified by exposure (MAP < 65 burden ≥ 60 mmHg·min). Values are median (IQR) or *n* (%).

Variable	Untreated (*n* = 2320)	Treated (*n* = 406)
Age, yr—median (IQR)	61 (52–69)	63 (53–72)
BMI—median (IQR)	22.9 (20.7–25.1)	22.7 (20.6–25.0)
Male sex—*n* (%)	1343 (57.9%)	275 (67.7%)
Preoperative creatinine, mg/dL	0.81 (0.68–0.97)	0.82 (0.69–1.04)
CKD proxy: creatinine ≥ 1.4—*n* (%)	151 (6.5%)	39 (9.6%)
Preoperative hemoglobin, g/dL	13.0 (11.7–14.3)	12.1 (10.4–13.4)
Preoperative albumin, g/dL	4.20 (3.80–4.40)	3.90 (3.30–4.20)
Hypertension—*n* (%)	780 (33.6%)	168 (41.4%)
Diabetes mellitus—*n* (%)	292 (12.6%)	70 (17.2%)
Emergency operation—*n* (%)	271 (11.7%)	65 (16.0%)
AKI (KDIGO Stage 1+)—*n* (%)	124 (5.3%)	81 (20.0%)

**Table 2:** Average treatment effect estimators across exposure thresholds. RD, risk difference (treated − untreated, percentage points).

Threshold (mmHg·min)	P (A = 1)	Crude RD	G-comp RD	IPTW RD	Primary AIPW [95% CI]
≥30	26%	+8.89	+0.42	+4.59	+2.78 [+0.83, +4.09]
≥60	15%	+14.61	+0.72	+7.68	+3.00 [+0.84, +5.26]
≥120	8%	+22.43	+3.29	+15.14	+7.62 [+3.24, +11.48]

**Table 3:** Within-cohort head-to-head AUROC comparison for predicting postoperative AKI (test split, *n* = 818).

Score (Preoperative Only)	*n* Feat.	AUROC [95% CI]	Interpretation
Preoperative creatinine alone	1	0.580 [0.485, 0.685]	Weak
ASA-PS alone	1	0.741 [0.668, 0.806]	Existing score
Simple logit (cr + age + DM + ASA)	4	0.775 [0.697, 0.841]	4-feature plateau
Phase 3 CATE GBM	27	0.768 [0.683, 0.836]	No gain

## Data Availability

The VitalDB dataset is publicly available at https://vitaldb.net (accessed on 14 May 2026). The analysis code is available from the corresponding author upon reasonable request.

## Supplementary Materials

The following supporting information can be downloaded at https://www.mdpi.com/article/10.3390/jpm16070371/s1, Figure S1: Sensitivity analysis across MAP thresholds; Figure S2: Predicted versus true CATE scatter for the Phase 3 M1 model (pre-operative tabular gradient-boosted machine); Figure S3: ROC curves for detecting the top-10% high-CATE patient subset (those who benefit most from intraoperative MAP support); Table S1: Phase 2.A—Controlled-direct-effect sensitivity analysis; Table S2: Phase 2.B—Negative control outcome (NCO) analyses at exposure threshold 60 mmHg·min; Table S3: Phase 2.C—E-value sweep across exposure thresholds and adjustment sets; Table S4: Phase 2.D—Vasopressor-stratified AIPW; Table S5: Phase 3 CATE-prediction model comparison; Table S6: Phase 4.A/B—Temporal hold-out and leave-one-department-out (LOO) validation; Table S7: Top-12 GBM feature importances (Gini) for CATE prediction in the Phase 3 M1 model (pre-operative tabular gradient-boosted machine, baseline-only adjustment, 70% training split).

## References

[1] Prowle J.R., Forni L.G., Bell M., Chew M.S., Edwards M., Grams M.E., Grocott M.P., Liu K.D., McIlroy D., Murray P.T. (2021). Postoperative acute kidney injury in adult non-cardiac surgery: Joint consensus report of the Acute Disease Quality Initiative and PeriOperative Quality Initiative. Nat. Rev. Nephrol..

[2] Chertow G.M., Burdick E., Honour M., Bonventre J.V., Bates D.W. (2005). Acute kidney injury, mortality, length of stay, and costs in hospitalized patients. J. Am. Soc. Nephrol..

[3] Worrall R.E., Mughal S.J., Parekh D., Patel J.M., McNulty D., Bangash M.N. (2025). Novel biological risk factors for 7-day postoperative kidney injury in elective major non-cardiac surgery: A retrospective observational study. Anaesthesia.

[4] Sun L.Y., Wijeysundera D.N., Tait G.A., Beattie W.S. (2015). Association of intraoperative hypotension with acute kidney injury after elective noncardiac surgery. Anesthesiology.

[5] Salmasi V., Maheshwari K., Yang D., Mascha E.J., Singh A., Sessler D.I., Kurz A. (2017). Relationship between intraoperative hypotension, defined by either reduction from baseline or absolute thresholds, and acute kidney and myocardial injury after noncardiac surgery: A retrospective cohort analysis. Anesthesiology.

[6] Bijker J.B., van Klei W.A., Kappen T.H., van Wolfswinkel L., Moons K.G.M., Kalkman C.J. (2007). Incidence of intraoperative hypotension as a function of the chosen definition: Literature definitions applied to a retrospective cohort using automated data collection. Anesthesiology.

[7] Sessler D.I., Bloomstone J.A., Aronson S., Berry C., Gan T.J., Kellum J.A., Plumb J., Mythen M.G., Grocott M.P., Edwards M.R. (2019). Perioperative Quality Initiative consensus statement on intraoperative blood pressure, risk, and outcomes for elective surgery. Br. J. Anaesth..

[8] Saugel B., Sander M., Katzer C., Hahn C., Koch C., Leicht D., Markmann M., Schneck E., Flick M., Kouz K. (2025). Association of intraoperative hypotension and cumulative norepinephrine dose with postoperative acute kidney injury in patients having noncardiac surgery: A retrospective cohort analysis. Br. J. Anaesth..

[9] Kheterpal S., Tremper K.K., Englesbe M.J., O’Reilly M., Shanks A.M., Fetterman D.M., Rosenberg A.L., Swartz R.D. (2007). Predictors of postoperative acute renal failure after noncardiac surgery in patients with previously normal renal function. Anesthesiology.

[10] Lee H.C., Park Y., Yoon S.B., Yang S.M., Park D., Jung C.W. (2022). VitalDB, a high-fidelity multi-parameter vital signs database in surgical patients. Sci. Data.

[11] von Elm E., Altman D.G., Egger M., Pocock S.J., Götzsche P.C., Vandenbroucke J.P., STROBE Initiative (2007). The Strengthening the Reporting of Observational Studies in Epidemiology (STROBE) statement: Guidelines for reporting observational studies. Lancet.

[12] Collins G.S., Moons K.G., Dhiman P., Riley R.D., Beam A.L., Van Calster B., Ghassemi M., Liu X., Reitsma J.B., Van Smeden M. (2024). TRIPOD+AI statement: Updated guidance for reporting clinical prediction models that use regression or machine learning methods. BMJ.

[13] (2012). Kidney Disease: Improving Global Outcomes (KDIGO) Acute Kidney Injury Work Group. KDIGO Clinical Practice Guideline for Acute Kidney Injury. Kidney Int. Suppl..

[14] Bang H., Robins J.M. (2005). Doubly robust estimation in missing data and causal inference models. Biometrics.

[15] Funk M.J., Westreich D., Wiesen C., Stürmer T., Brookhart M.A., Davidian M. (2011). Doubly robust estimation of causal effects. Am. J. Epidemiol..

[16] Lipsitch M., Tchetgen Tchetgen E., Cohen T. (2010). Negative controls: A tool for detecting confounding and bias in observational studies. Epidemiology.

[17] VanderWeele T.J., Ding P. (2017). Sensitivity analysis in observational research: Introducing the E-value. Ann. Intern. Med..

[18] Wager S., Athey S. (2018). Estimation and inference of heterogeneous treatment effects using random forests. J. Am. Stat. Assoc..

[19] Athey S., Tibshirani J., Wager S. (2019). Generalized random forests. Ann. Stat..

[20] Vickers A.J., Elkin E.B. (2006). Decision curve analysis: A novel method for evaluating prediction models. Med. Decis. Mak..

[21] Penev Y., Ruppert M.M., Bilgili A., Li Y., Habib R., Dozic A.V., Small C., Adiyeke E., Ozrazgat-Baslanti T., Loftus T.J. (2024). Intraoperative hypotension and postoperative acute kidney injury: A systematic review. Am. J. Surg..

[22] Futier E., Lefrant J.Y., Guinot P.G., Godet T., Lorne E., Cuvillon P., Bertran S., Leone M., Pastene B., Piriou V. (2017). Effect of individualized vs standard blood pressure management strategies on postoperative organ dysfunction among high-risk patients undergoing major surgery: A randomized clinical trial. JAMA.

[23] Saugel B., Fletcher N., Gan T.J., Grocott M.P., Myles P.S., Sessler D.I., Auzinger G., Chappell D., Edwards M., Forni L.G. (2024). PeriOperative Quality Initiative (POQI) international consensus statement on perioperative arterial pressure management. Br. J. Anaesth..

