# Supplementary material for: Causal Effect and Personalization of Intraoperative Hypotension Burden on Postoperative Acute Kidney Injury: A Doubly Robust Analysis of the VitalDB Cohort
Source: J Pers Med. 2026 Jul 10;16(7):371. doi: 10.3390/jpm16070371 (PMC13413355; doi:10.3390/jpm16070371)
Supplement: Supplementary file 1 [file jpm-16-00371-s001.zip › jpm-4401379-supplementary.pdf]

Supplementary Material

Causal inference and personalization framework for intraoperative hypotension and post-operative acute kidney injury in the VitalDB cohort

Overview

This supplementary file contains two supplementary figures (S1–S2) and seven supplementary tables (S1–S7) supporting the main manuscript. Phase 2 (sensitivity analyses) is supported by Supplementary Tables S1–S4, covering the controlled-direct-effect comparison, negative control outcomes, E-value sweep, and vasopressor-stratified analysis. Phase 3 (CATE personalization) is supported by Supplementary Figures S1–S2 and Supplementary Tables S5 and S7, covering predicted-versus-true CATE scatter, top-decile ROC, model-variant comparison, and feature importance. Phase 4 (internal validation) is supported by Supplementary Table S6, covering temporal and leave-one-department-out validation.

Contents

Supplementary Figures

Figure S1. Sensitivity forest plot across thresholds (AIPW, CDE, vasopressor strata, NCO)

Figure S2. Predicted vs true CATE scatter (M1 tabular GBM)

Figure S3. ROC for top-10% high-CATE patient detection

Supplementary Tables

Table S1. Controlled-direct-effect sensitivity vs primary total effect

Table S2. Negative control outcome (NCO) results

Table S3. E-value sweep across thresholds and adjustment sets

Table S4. Vasopressor-stratified AIPW results

Table S5. Phase 3 CATE prediction model comparison

Table S6. Phase 4 temporal and department leave-one-out validation

Table S7. Top-12 GBM feature importances for CATE prediction

Supplementary Figures

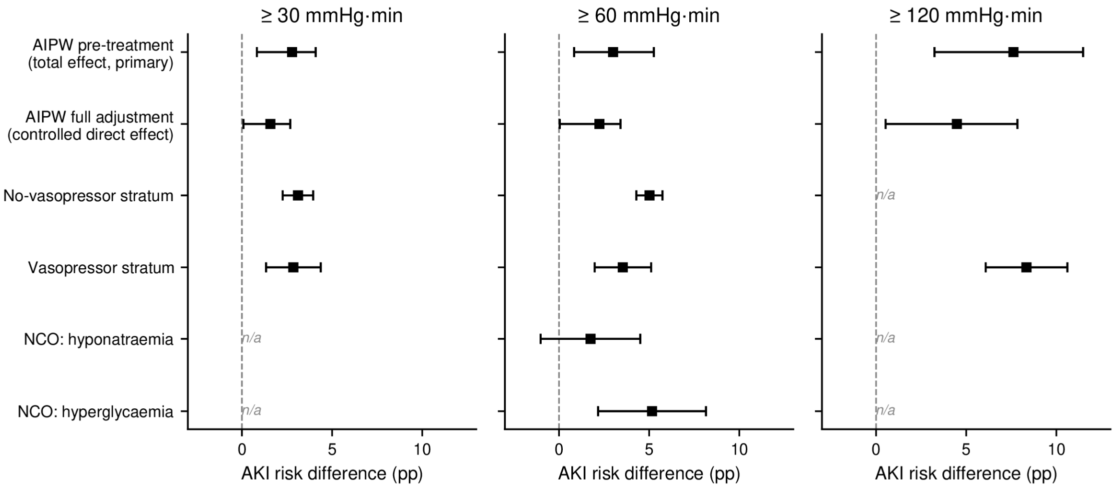

Supplementary Figure S1. Sensitivity analysis across MAP thresholds (≥30, ≥60, ≥120 mmHg·min): AIPW pre-treatment total effect (primary), AIPW full adjustment (controlled direct effect), no-vasopressor and vasopressor strata, and negative-control outcomes. Points are AKI risk differences (percentage points) with 95% confidence intervals; n/a denotes strata with insufficient events.

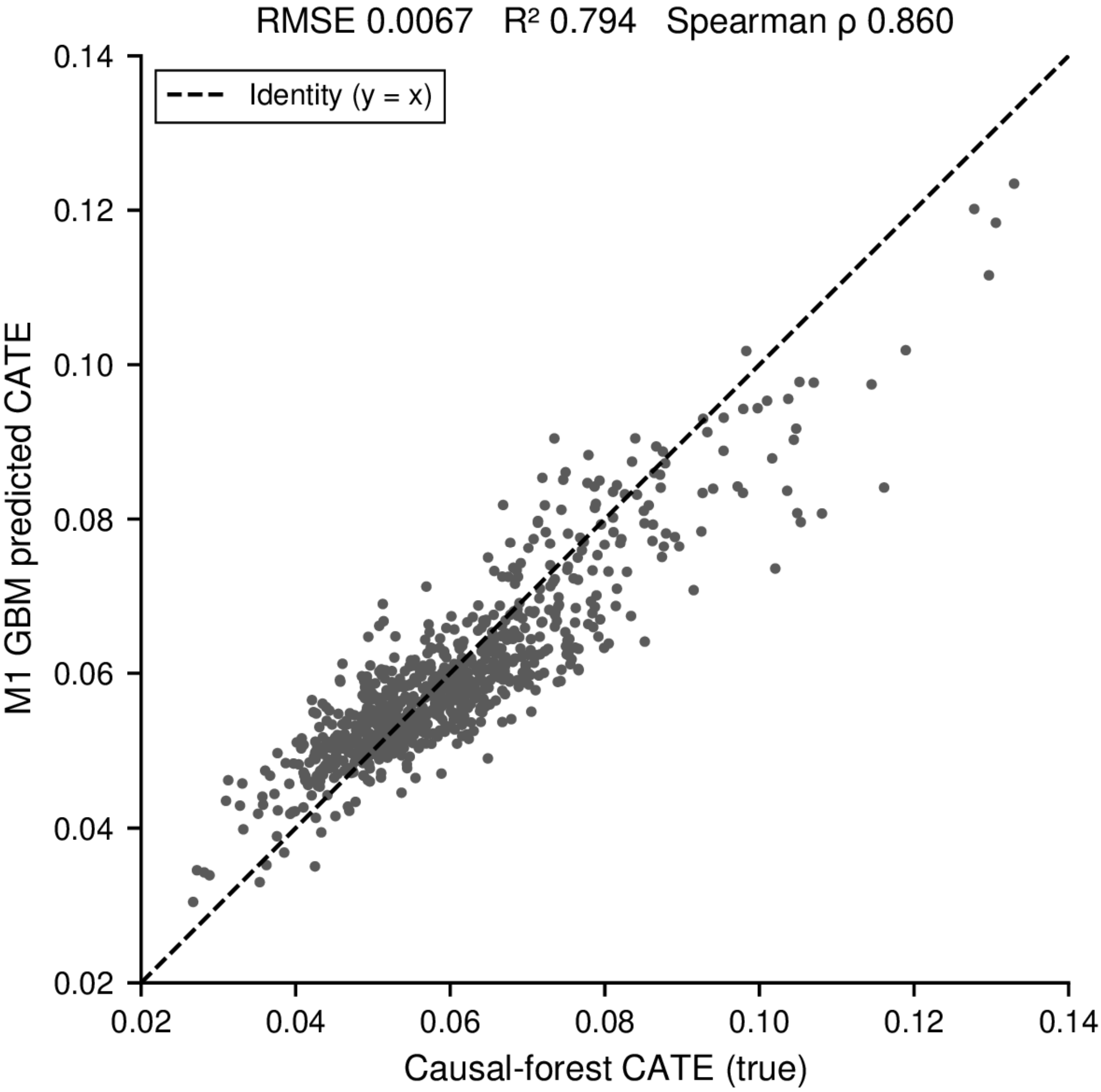

Supplementary Figure S2. Predicted versus true CATE scatter for the Phase 3 M1 model (pre-operative tabular gradient-boosted machine). Each point is a held-out test patient (n = 818). The dashed line denotes the identity (y = x). RMSE 0.0067, R^2 0.794, Spearman ρ 0.860. The model captures the CATE structure across the +0.03 to +0.13 range.

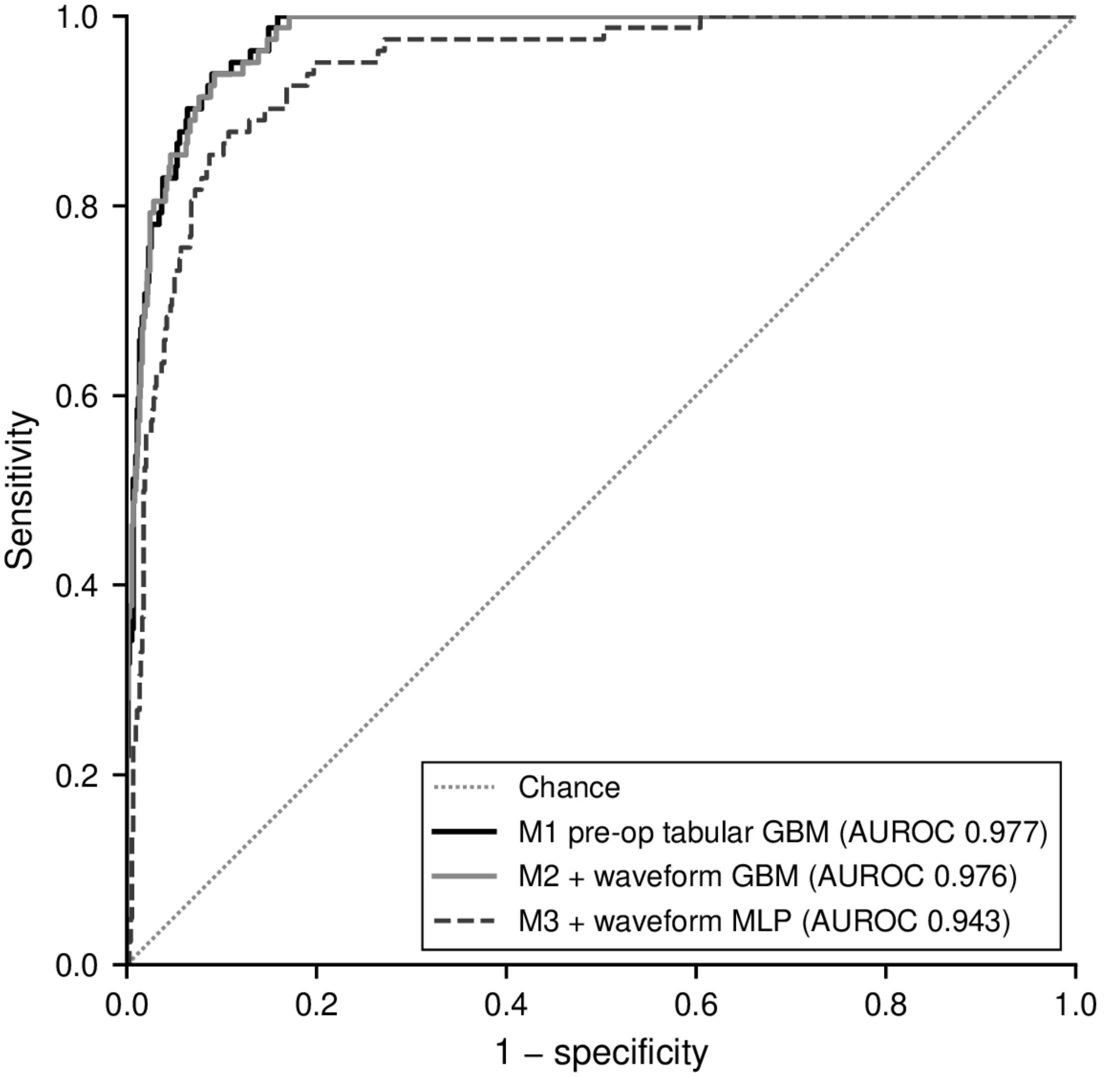

Supplementary Figure S3. ROC curves for detecting the top-10% high-CATE patient subset (those who benefit most from intraoperative MAP support). M1 pre-operative tabular GBM and M2 +waveform GBM achieve essentially identical performance (AUROC 0.977 vs 0.976); the M3 +waveform multi-layer perceptron underperforms (0.943), consistent with overfitting on a sample of this size.

Supplementary Tables

Supplementary Table S1. Phase 2.A — Controlled-direct-effect sensitivity analysis. The pre-specified primary AIPW (pre-treatment covariates only) estimates the total causal effect; the full-adjustment model, which additionally conditions on the intraoperative mediators (fluid volume, vasopressor use, anesthesia duration), estimates the controlled direct effect. Bootstrap 95% confidence intervals are shown for both. The difference (Δ) approximates the portion of the total effect mediated through intraoperative fluid and vasopressor management, and is larger at higher exposure thresholds.

Threshold	Primary AIPW — total effect [bootstrap 95% CI]	Controlled direct effect AIPW (full adj.) [bootstrap 95% CI]	Δ (mediated through intraop

			fluids/vasopressor)
≥ 30	+2.78 [+0.83, +4.09]	+1.58 [+0.08, +2.68]	+1.20 pp
≥ 60	+3.00 [+0.84, +5.26]	+2.24 [+0.04, +3.41]	+0.76 pp
≥ 120	+7.62 [+3.24, +11.48]	+4.48 [+0.53, +7.84]	+3.14 pp

Supplementary Table S2. Phase 2.B — Negative control outcome (NCO) analyses at exposure threshold 60 mmHg·min. Hyponatremia (no plausible direct link to MAP burden) shows a null association, supporting the absence of generalised residual confounding. Hyperglycemia shows a positive association, interpreted as evidence of shared upstream peri-operative confounders (stress response, fluid and dextrose management) rather than as invalidating the primary causal estimate.

NCO outcome	Mechanism	n (with obs)	AIPW RD (t=60) [95% CI]	Verdict
hyponatremia	Fluid / SIADH	2347	+1.75 pp [-1.02, +4.51]	Pass (CI crosses 0)
hyperglycemia	Stress + dextrose	1488	+5.16 pp [+2.17, +8.15]	Fail (dose-response)

Supplementary Table S3. Phase 2.C — E-value sweep across exposure thresholds and adjustment sets. An E-value greater than 2 indicates that an unmeasured confounder would need to be associated with both exposure and outcome, on the risk-ratio scale, by more than two-fold above and beyond the measured covariates, to fully nullify the observed association. The ≥120 mmHg·min threshold exceeds this threshold at both point estimate and CI-lower bound.

Threshold	Crude E-value	AIPW full E-value (CI-low)	AIPW baseline-only E-value (CI-low)
≥ 30	4.87	1.71 (1.16)	2.08 (1.65)
≥ 60	6.93	1.92 (1.13)	2.15 (1.70)
≥ 120	9.20	2.57 (2.09)	3.44 (2.82)

Supplementary Table S4. Phase 2.D — Vasopressor-stratified AIPW. AIPW estimated separately within the no-vasopressor and vasopressor strata, as a G-formula proxy for addressing treatment–confounder feedback. The positive association holds in both strata, arguing against vasopressor-mediated confounding as a sole explanation.

Threshold	No-vasopressor (n = 1003)	Vasopressor (n = 1723)
≥ 30	+3.11 pp [+2.26, +3.95]	+2.85 pp [+1.34, +4.37]
≥ 60	+5.02 pp [+4.29, +5.74]	+3.54 pp [+1.98, +5.11]
≥ 120	(too sparse)	+8.34 pp [+6.08, +10.61]

Supplementary Table S5. Phase 3 CATE-prediction model comparison. M1 uses 27 pre-operative tabular features (gradient-boosted machine). M2 extends M1 with 20 intraoperative waveform-derived features (also GBM). M3 uses the same combined 47-feature set with a multi-layer perceptron

architecture. Adding intraoperative waveform features provides no measurable improvement over the pre-operative tabular baseline. The AUROC columns quantify recovery of the causal-forest-derived CATE target (identification of the top-decile and CKD high-CATE subsets), not prediction of AKI occurrence, which is reported in main-text Table 3.

**Model**	**n_features**	**RMSE**	**Spearman ρ**	**R^2**	**AUROC top-10%**	**AUROC CKD**
M1 Preop tabular GBM	27	0.0067	0.860	0.794	0.977	0.848
M2 +Waveform GBM	47	0.0068	0.859	0.789	0.976	0.852
M3 +Waveform MLP (NN)	47	0.0097	0.710	0.572	0.943	0.833

Supplementary Table S6. Phase 4.A/B — Temporal hold-out and leave-one-department-out (LOO) validation. Performance is preserved across both validation regimes, supporting stability under temporal drift and across surgical contexts.

**Validation strategy**	**n_train**	**n_test**	**RMSE**	**Spearman ρ**	**AUROC top-10%**
Temporal (first 70% caseid → last 30%)	1908	818	0.0065	0.858	0.987
Leave-out: General surgery	994	1732	0.0089	0.811	0.938
Leave-out: Thoracic surgery	1864	862	0.0065	0.815	0.976
Leave-out: Urology	2646	80	0.0057	0.798	0.936
Leave-out: Gynecology	2674	52	0.0064	0.858	0.982

Supplementary Table S7. Top-12 GBM feature importances (Gini) for CATE prediction in the Phase 3 M1 model (pre-operative tabular gradient-boosted machine, baseline-only adjustment, 70% training split). Pre-operative albumin (0.253) and creatinine (0.173) together account for over 40% of total feature importance, confirming a CKD-centric basis for personalized IOH–AKI susceptibility. The remaining importance is distributed across hemoglobin, platelet count, weight, and other pre-operative laboratory variables.

**Rank**	**Feature**	**Importance (Gini)**
1	preop_alb	0.253
2	preop_cr	0.173
3	preop_hb	0.080
4	preop_plt	0.074
5	weight	0.060

6	preop_na	0.058
7	preop_bun	0.048
8	age	0.041
9	emop	0.039
10	preop_aptt	0.038
11	height	0.037
12	preop_pt	0.033
